# Arm-to-retina time predicts visual outcome of anti-vascular endothelial growth factor treatment for macular edema due to central retinal vein occlusion

**DOI:** 10.1038/s41598-022-06281-w

**Published:** 2022-02-09

**Authors:** Tatsuhiko Takei, Norihiro Nagai, Kishiko Ohkoshi, Yoko Ozawa

**Affiliations:** 1 Department of Ophthalmology, St. Luke’s International Hospital, 9-1 Akashi-cho, Chuo-ku, Tokyo, 104-8560 Japan; 2 Laboratory of Retinal Cell Biology, St. Luke’s International University, 9-1 Akashi-cho, Chuo-ku, Tokyo, 104-8560 Japan; 3 Laboratory of Retinal Cell Biology, Department of Ophthalmology, Keio University School of Medicine, 35 Shinanomachi, Shinjuku-ku, Tokyo, 160-8582 Japan; 4 Department of Ophthalmology, Keio University School of Medicine, 35 Shinanomachi, Shinjuku-ku, Tokyo, 160-8582 Japan; 5 Laboratory of Retinal Cell Biology, Department of Ophthalmology, St. Luke’s International University and Hospital, Tokyo, 104-8560 Japan

**Keywords:** Eye diseases, Predictive markers

## Abstract

To explore the factors associated with best-corrected visual acuity (BCVA) after anti-vascular endothelial growth factor (anti-VEGF) treatment for macular edema secondary to central retinal vein occlusion (CRVO). We retrospectively reviewed the medical charts of 22 eyes of 22 treatment-naïve patients with CRVO diagnosed between September 2014 and December 2020. They received anti-VEGF treatment and follow-up for > 12 months. Mean patient age was 64.3 years; 13 (59.1%) were men. Eyes with baseline arm-to-retina (AR) time ≥ 16 s had better BCVA at 12 months (adjusted for baseline BCVA and age; B, − 0.658; 95% confidence interval − 1.058 to − 0.257; P = 0.003), greater mean BCVA change (P = 0.006), lower frequency of residual macular edema at 12 months (P = 0.026) and recurrent and/or unresolved macular edema during 12 months (P = 0.046), and higher frequency of reduction in central retinal thickness ≥ 150 μm at 1 and 12 months (both P = 0.046). Delayed AR time was associated with a better visual outcome and macular edema improvement in CRVO after anti-VEGF treatment regardless of initial BCVA and age. Our results may help understand the pathogenesis and predict the visual prognosis of patients before anti-VEGF therapy initiation.

## Introduction

Prognosis of macular edema secondary to central retinal vein occlusion (CRVO) has been shown to improve with anti-vascular endothelial growth factor (anti-VEGF) treatment in clinical trials and real-world clinical settings^1^. However, the visual prognosis of each individual varies, most probably due to the differences in pathogenesis^2^. Here we analyze the factors that can affect visual prognosis to enhance the understanding of the pathogenesis of this condition and help predict the prognosis in the daily clinical setting.

Apart from age^3^, the risk factors for retinal vein occlusion (RVO) are hypertension^4–6^ and high hematocrit value, which is related to blood viscosity^5^; the latter two factors are independent of age and sex^5^. Damage to the retinal vessel wall due to atherosclerosis and compression alters rheologic properties in the adjacent central vein, contributing to stasis, hypercoagulability, thrombosis, and thus occlusion^7^. Although CRVO is a rare condition, in approximately 0.2% of the individuals over the age of 40 years in Japan^5^, the occlusion is at or proximal to the lamina cribrosa of the optic nerve where the central retinal vein exits the eye, and the entire retina is affected. Although gradual development of neovascularization related to retinal ischemia can also cause visual impairment^8^, macular edema due to breakdown of the blood–retina barrier in the macular area is the direct cause of visual loss^9^.

Previous analyses have focused on disrupted optical coherence tomography (OCT) findings of the external limiting membrane, ellipsoid zone, and interdigitation zone as predictors of visual outcome after treatment of macular edema due to RVO^10–12^. However, these factors can also affect the initial best-corrected visual acuity (BCVA), which is the major predictive factor for visual prognosis in RVO, including CRVO^3^, and thus could be confounding factors. Reports have also shown that a longer duration from onset to treatment is associated with a worse prognosis in CRVO^1^. However, this may also be related to initial BCVA and OCT findings.

Here we focus on arm-to-retina (AR) time, measured from the administration of fluorescein into the antecubital vein until it becomes visible in the retinal arteries during fluorescein angiography (FA) recording^13^. The AR time is related to arteriosclerosis^14^, coronary slow-flow^15^, as well as carotid artery occlusion^16^, and systemic hemodynamics. Thus, to analyze the impact of AR time in CRVO in which hypertension and blood hyperviscosity are the risk factors^5^, would be of value. In addition, while previous reports have often evaluated BCVA change^17–19^, the final BCVA value is also important for lifestyle management after treatment. In this study, we analyzed the data of patients with CRVO to identify factors that affect visual outcome. The study will help to understand the pathogenesis of CRVO and facilitate obtaining appropriate informed consent regarding the visual outcome of the treatment from patients in the daily clinic.

## Results

Twenty-two eyes of 22 patients were analyzed (Table 1). The mean age of the patients was 64.3 ± 18.0 (range 25–86) years, and 13 patients (59.1%) were men. Mean LogMAR BCVA was 0.678 ± 0.56 (range 0.000 to counting fingers), and mean central retinal thickness (CRT) was 465.36 ± 137.8 (range 267–764) μm at baseline. Mean AR time in FA was 15.85 ± 4.6 (range 9.1–32.1) s, and mean systolic and diastolic blood pressures were 138.0 ± 15.2 (range 104–166) and 76.9 ± 11.4 (range 46–97) mmHg, respectively. Ischemic CRVO was observed in 7 eyes (31.8%).

**Table 1:** Baseline characteristics.

Eyes	22
Age (years [range])	64.3 ± 18.0 (25–86)
Sex (men [%])	13 (59.1)
Best-corrected visual acuity (LogMAR [range])	0.678 ± 0.56 (0.000 to counting fingers)
Central retina thickness (μm [range])	465.36 ± 137.8 (267–764)
Arm to retina time (sec [range])	15.85 ± 4.6 (9.1–32.1)
Systolic blood pressure (mmHg [range])	138.0 ± 15.2 (104–166)
Diastolic blood pressure (mmHg [range])	76.9 ± 11.4 (46–97)
Intraocular pressure (mmHg [range])	13.5 ± 3.1 (10–23)
Ischemic CRVO (eyes [%])	7 (31.8)

Data are represented as the mean ± SD.

**Figure 1:**
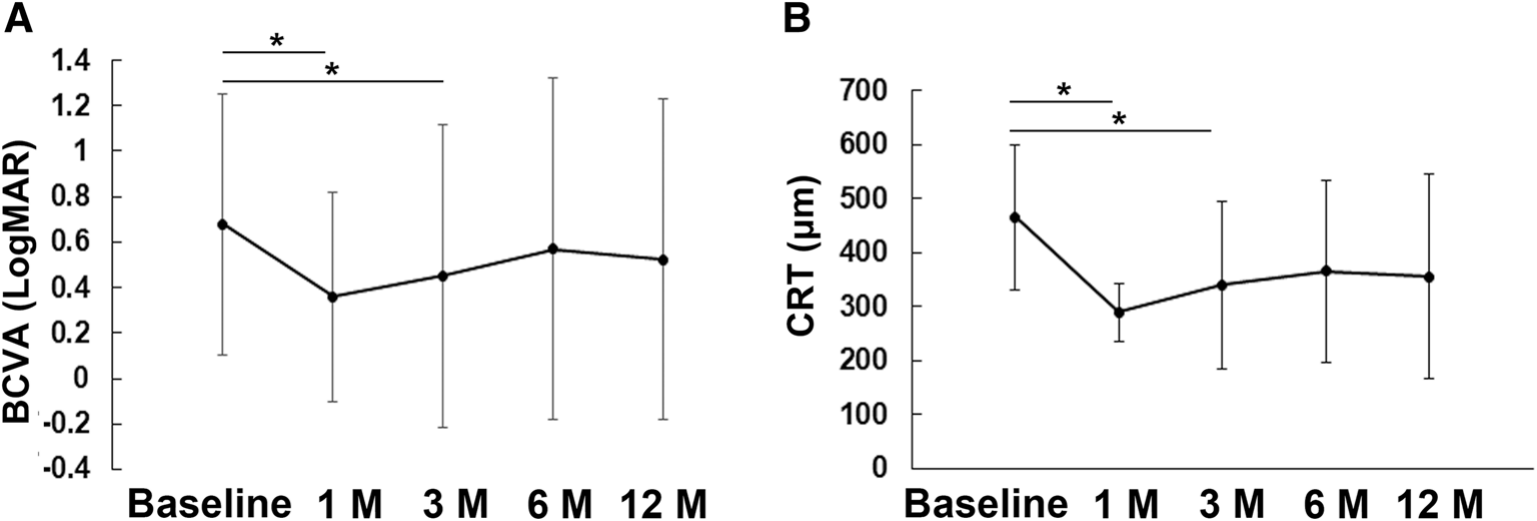
Mean best-corrected visual acuity (BCVA) and central retinal thickness (CRT) at each time-point after initial anti-vascular endothelial growth factor treatment. Data are represented as the mean ± standard deviation. The values at 1 and 3 months post-treatment were improved compared with those at baseline; however, this improvement was not observed at the 6 and 12 month time-points. Generalized mixed model analysis, *P < 0.05.

Overall, mean BCVA at 1 month and 3 months after the initial anti-VEGF treatment was 0.358 ± 0.717 (P < 0.001) and 0.451 ± 0.67 (P = 0.020), and mean CRT at the same time-points was 288.96 ± 53.3 μm (P = 0.001) and 339.41 ± 155.0 μm (P = 0.027), respectively; BCVA and CRT were significantly improved compared to those at baseline at these time-points (Fig. 1). However, no significant improvements in mean BCVA (0.523 ± 0.70, range: − 0.176 to hand motion; P = 0.22) or mean CRT (354.9 ± 188.8 μm, range 182–812 μm, P = 0.73) compared to the baseline values were noted at 12 months after the initial anti-VEGF treatment. There were also no significant improvements at 6 months (mean BCVA, 0.570 ± 0.75, P = 0.62; mean CRT, 366.0 ± 168.2 μm, P = 0.14).

As the outcome values at 12 months varied from individual to individual, we investigated the factors associated with good visual outcome. We found that eyes with initial AR time ≥ 16 s in FA that was longer than the normal range of AR time (10–15 s)^20^ had better BCVA at 12 months after adjusting for baseline BCVA and age (B, − 0.658; 95% confidence interval − 1.058 to − 0.257; P = 0.003) (Table 2). Sex, initial CRT, initial intraocular pressure, actual value of AR time, initial systolic and diastolic blood pressures, and additional pan-retinal photocoagulation treatment during 12 months of follow-up did not significantly affect the visual outcome.

**Table 2:** Factors associated with visual outcome 12 months after initial treatment.

Variables	B	95% confidence interval	P
Arm to retina time ≥ 16 s	− 0.658	− 1.058 to − 0.257	0.003**

Forced input analysis adjusted for age and initial best-corrected visual acuity. The selected factors were sex, initial central retinal thickness, initial intraocular pressure, actual value of AR time, initial systolic blood pressure, initial diastolic blood pressure, and additional photocoagulation treatment during 12 months of follow-up.

**P < 0.01.

Eyes with AR time ≥ 16 s exhibited a significantly better BCVA change (P = 0.006), and a trend of greater CRT reduction at 12 months (P = 0.069) (Table 3). More importantly, a significantly smaller number of eyes with AR time ≥ 16 s still exhibited macular edema at 12 months (P = 0.026)—only 10% of eyes with AR time ≥ 16 s exhibited macular edema, while this percentage was 58.3% in the other group. We also analyzed whether the eyes with AR time ≥ 16 s had a lower risk of recurrent or unresolved macular edema during 12 months. Our results showed that only 30% of the eyes with AR time ≥ 16 s had recurrent or unresolved edema during the entire 12-month period, while this percentage was 75% in the other group (P = 0.046). In addition, reduction in CRT ≥ 150 μm both at 1 and 12 months after initial anti-VEGF treatment was achieved more frequently in eyes with AR time ≥ 16 s (both P = 0.046), indicating that these eyes had better responsiveness to anti-VEGF treatment.

**Table 3:** Comparison of the AR time < 16 s and AR time ≥ 16 s groups.

	AR time < 16 s	AR time ≥ 16 s	P
	n = 12	n = 10	
Age (years [range])	64.0 ± 17.9 (25–81)	64.7 ± 18.6 (35–86)	1.000
Sex (men [%])	5 (45.5)	8 (72.7)	0.771
Initial BCVA (LogMAR])	0.591 ± 0.56	0.782 ± 0.60	0.381
Change in BCVA at 12 months	0.181 ± 0.48	− 0.466 ± 0.42	0.006*
Initial CRT (μm)	452.3 ± 127.6	481.1 ± 146.9	0.674
Change in CRT at 12 months (μm)	− 16.6 ± 271.8	− 223.1 ± 187.2	0.069
Initial ischemic CRVO (eyes [%])	4 (33.3)	3 (30.0)	0.616
Initial macular ischemia (eyes [%])	2 (16.7)	0 (0.0)	0.286
Initial SRF (eyes [%])	6 (50.0)	8 (80.0)	0.340
Presence of macular edema at 12 months (eyes [%])	7 (58.3)	1 (10.0)	0.026*
Recurrent or unresolved macular edema during 12 months (eyes [%])	9 (75.0)	3 (30.0)	0.046*
Reduction in CRT ≥ 150 μm at 1 month (eyes [%])	3 (25.0)	7 (70.0)	0.046*
Reduction in CRT ≥ 150 μm at 12 months (eyes [%])	3 (25.0)	7 (70)	0.046*
Systolic blood pressure (mmHg)	138.3 ± 18.1	137.6 ± 12.3	0.771
Diastolic blood pressure (mmHg)	77.4 ± 13.6	76.3 ± 8.2	0.628
Intraocular pressure (mmHg [range])	13.4 ± 2.5	13.6 ± 3.9	0.817
History of glaucoma (eyes [%])	0 (0.0)	1 (10.0)	0.455
Number of injections in 12 months	3.92 ± 2.1	4.30 ± 2.1	0.674
Number of injections until the first resolution of macular edema	1.50 ± 1.0	1.20 ± 0.42	0.653
Additional treatment with photocoagulation (%)	8 (72.7)	8 (80.0)	0.646

Data are represented as the mean ± SD. The Mann–Whitney U test or Fishers' exact test (one-sided) were used for comparison.

*AR time* arm-to-retina time, *BCVA* best-corrected visual acuity, *CRT* central retinal thickness, *CRVO* central retinal vein occlusion, *SRF* subretinal fluid.

*P < 0.05.

**Figure 2:**
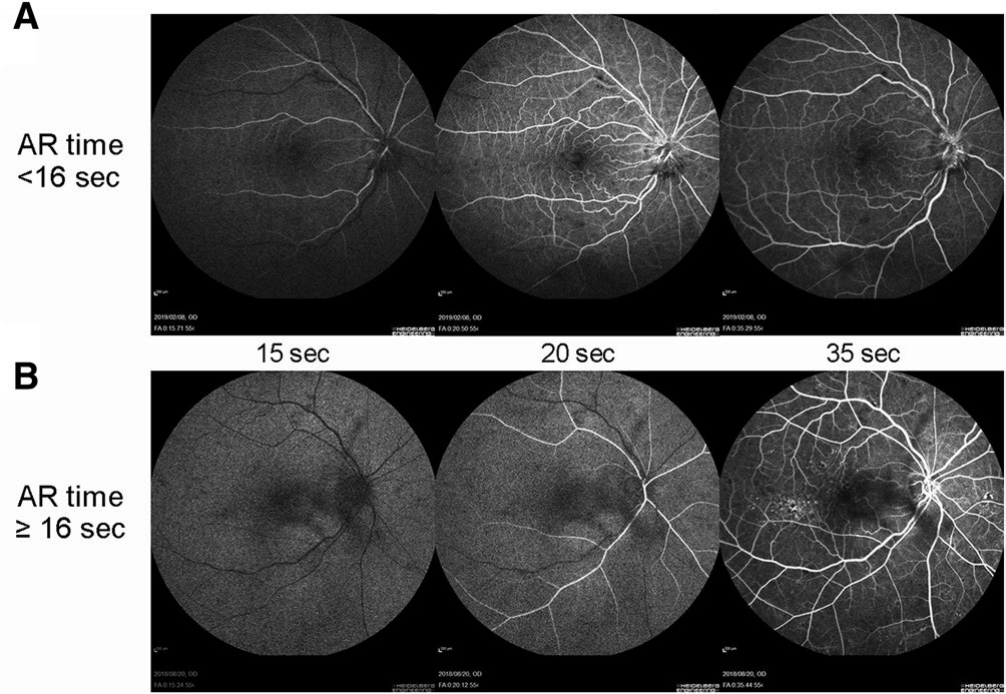
Representative images of fluorescein angiography of eyes with arm-to-retina (AR) time < 16 s and ≥ 16 s. (**A**) Images of the eye of an 80-year-old woman with AR time 13.3 s (best-corrected visual acuity [BCVA], 0.398 at 12 months; increase in central retinal thickness (CRT), 393 μm at 12 months compared with baseline). (**B**) Images of the eye of a 86-year-old woman with AR time 17.7 s (BCVA, 0.000 at 12 months; reduction in CRT, 186 μm at 12 months compared with baseline). FA images 15, 20, and 35 s after the time point that the dye reached the injection inserted point are shown.

Representative FA images of eyes with AR time ≥ 16 s and < 16 s are shown in Fig. 2.

## Discussion

The study showed that the outcome of anti-VEGF treatment varied from individual to individual in patients with CRVO-related macular edema. Eyes with AR time ≥ 16 s exhibited better BCVA 12 months after anti-VEGF treatment with or without pan-retinal photocoagulation. These eyes exhibited residual or recurrent macular edema less frequently at 12 months and experienced recurrence or absence of remission of macular edema less frequently during this period. The frequency of reduction in CRT ≥ 150 μm at 1 and 12 months compared to that at baseline was also higher.

The mean age of the CRVO patients in the current study was 64.3 years, which was similar to that in previous clinical trials on treatment-naïve patients, such as the CRUISE study (68 years)^18^, COPERNICUS study (66.3 years)^21^, and GALILEO study (61.5 years)^22^. A Japanese population-based cohort study, the Hisayama Study, also reported the mean age of patients with branch RVO and CRVO as 63.0 years^6,18^. The mean systolic blood pressure was 138 mmHg, close to the global hypertension practice guideline value of 140 mmHg^23^. Blood pressure over 130 mmHg is defined as elevated blood pressure, a known risk factor for hypertension in Japan^24^. High-normal blood pressure (systolic, 130–139/85–89 mmHg) exhibited an odds ratio of 6.81 with regard to the prevalence of RVO in the Hisayama Study^5^.

It is reported that initial BCVA as well as initial OCT findings, which may reflect the BCVA, are related to visual outcome^10–12^. However, we found that AR time ≥ 16 s in FA at baseline was associated with better BCVA 12 months after anti-VEGF treatment adjusting for initial BCVA and age. A longer AR time is related to worse vascular circulation^14–16^, and AR time is prolonged in combined artery and vein occlusion^25^. However, in the absence of significant artery occlusion, as shown in the current study, longer AR time was advantageous for better visual outcome at 12 months.

This may be because residual macular edema at 12 months was less frequently observed in patients who exhibited AR time ≥ 16 s at baseline, as shown in the current study. The patients more frequently achieved resolution of the macular edema after the treatment and less frequently showed recurrent macular edema after the resolution during the follow-up period. Previous reports showed that recurrent macular edema is related to worse visual prognosis in RVO^26,27^. Thus, effective resolution of macular edema may have contributed to the better visual outcome in the patients with AR time ≥ 16 s. In fact, patients with AR time ≥ 16 s at baseline showed a reduction in CRT ≥ 150 μm 1 month after initial anti-VEGF treatment, suggesting that these patients responded well to the anti-VEGF treatment.

Anti-VEGF treatment is believed to improve the vascular obstruction related to inflammatory cells, termed leukostasis^9^. Given that patients with AR time ≥ 16 s at baseline had better response to anti-VEGF treatment, they may have had a major influence of leukostasis. The scenario is consistent with the fact that AR time was correlated with the mean blur rate, which represents blood flow velocity, measured using laser speckle flowgraphy at the optic nerve head in patients with RVO^14^. Thus, longer AR time is related to high velocity, which may be at least in part due to leukostasis. Taken together, patients with AR time ≥ 16 s at baseline may have had a good response to anti-VEGF therapy after leukostasis resolution by VEGF inhibition, and resulting blood flow recovery. Moreover, it has been reported that macular edema recurrence is related to sustained VEGF expression^9^, and suppression of leukostasis may have reduced chronic relative ischemia in the retinal tissue, leading to downregulation of VEGF expression from the retina. In contrast, patients with shorter AR time could have been more influenced by mechanistic occlusion related to vascular anatomy and arteriosclerosis, and less by leukostasis, although further studies are required to clarify these aspects.

The limitations of the current study included a retrospective design and a relatively small sample size; however, considering that the prevalence of CRVO (0.2% in individuals over 40 years of age) is 10 times lower than that of branch RVO^5,6^ (2% as reported in the Hisayama Study^5^), the sample size was considerable for a single-center analysis. Nevertheless, actual AR time did not have a statistically significant effect on the visual outcome, most likely due to the small sample size. We included patients who received either or both ranibizumab and aflibercept therapy and laser therapy during the 12 month study period. However, this was similar to most previous clinical trials involving rescue laser therapies for patients^17–19^. Laser therapy also reduces VEGF expression, most likely by reducing oxygen demand and/or VEGF producing retinal cell number; the treatment concept is similar to that of anti-VEGF therapy in this regard. Besides, laser therapy during the 12 months of follow-up was not found to be associated with visual outcome in the current study. Because informed consent was necessary for each re-injection, patients could refuse the re-treatment, however, we confirmed that retreatment had been performed properly at most times based on the criterion, if follow-up OCT showed exudative changes.

Visual outcome and BCVA at month 12 varied from patient to patient. This is consistent with previous reports on natural progression^8^ and anti-VEGF treatments^3^, and could be related to the differences in the pathogenesis of CRVO. AR time ≥ 16 s was associated with better visual outcome regardless of initial BCVA and age, which may help us to better understand one aspect of the pathogenesis of CRVO, as discussed above. In addition, clinicians may refer to the AR time to explore the pathological condition of the individual patients, and to better predict visual outcome, which would facilitate obtaining informed consent from patients before initiating anti-VEGF therapy in daily practice. Nevertheless, further studies are warranted to validate the results of this study.

## Methods

This retrospective study adhered to the tenets of the Declaration of Helsinki and was approved by the St. Luke’s International University Ethics Committee (approval number: 20-R048).

### Patients

The analyses were based on a detailed medical chart review of 22 eyes of 22 patients. All patients were treatment-naïve and were diagnosed with CRVO at the Vitreo-Retina Division Clinic of the Department of Ophthalmology at St. Luke’s International Hospital in Tokyo, Japan, between September 2014 and December 2020 and treated and followed-up for more than 12 months. Patients without FA records at baseline or had undergone prior treatment for CRVO, including anti-VEGF therapy, steroid therapy, intraocular surgery in the previous 3-month period, and laser photocoagulation, were excluded. Informed consent has been obtained from the participants.

### Eye examinations

All patients underwent BCVA measurement based on refraction tests, slit-lamp examinations, and binocular indirect ophthalmoscopy after pupil dilation with 0.5% tropicamide at each time-point. Data at baseline and 1, 3, 6, and 12 months after initial anti-VEGF treatment were analyzed.

### FA

For FA, we injected 10 mL containing 500 mg sodium fluorescein (Fluorescite; Novartis Pharma). The dyes were injected through an intravenous line over 10 s at the same rate for each patient and flushed through with sterile 5% glucose (Terumo Corporation, Tokyo Japan). Angiography was recorded from the time point that the dye reached the injection inserted point using a Heidelberg Spectralis HRA + OCT instrument (Heidelberg Engineering). The AR time was measured using the device equipped in the instrument. Fundus photographs for CRVO diagnosis were obtained using a Topcon TRC-50DX retinal camera (Topcon Corporation, Tokyo, Japan). Ischemic CRVO was defined as the presence of at least ten disc areas of retinal non-perfusion^28^. Macular ischemia was defined as the presence of retinal non-perfusion in the Early Treatment Diabetic Retinopathy Study grid center subfield^29^.

### OCT

OCT images were recorded at every follow-up visit using a Cirrus HD-OCT system (Zeiss, Oberkochen, Germany) or a Heidelberg Spectralis OCT system (Heidelberg Engineering GmbH, Dossenheim, Germany).

### Treatments

All patients were treatment naïve for CRVO and were initially treated either with intraocular injection of ranibizumab (0.5 mg [0.05 mL]) or aflibercept (2 mg [0.05 mL]). Re-injections were recommended if the OCT image and/or fundus examination showed evidence of any exudative changes in the macula, identified as macular edema and/or subretinal fluid at the time of the follow-up examinations, and performed under informed consent. Application of the drug was changed from ranibizumab to aflibercept in some of the patients. Pan-retinal laser photocoagulation, were provided at each doctor’s discretion.

### Statistical analyses

Data are expressed as the mean ± standard deviation (SD). Generalized mixed model analyses, forced input analyses adjusted for age and initial BCVA, the Mann–Whitney U test, and Fishers' exact test (one-sided) were performed using SPSS version 27.0 (SPSS Japan, Tokyo, Japan). P values < 0.05 were considered statistically significant.

### Ethics declarations

This retrospective study adhered to the tenets of the Declaration of Helsinki and was approved by the St. Luke’s International University Ethics Committee (approval number: 20-R048).

### Consent to participate/consent to publish

This retrospective study was approved by the St. Luke’s International University Ethics Committee (approval number: 20-R048).

## Data Availability

The datasets generated during and/or analyzed during the current study are available from the corresponding author on reasonable request.
